# Increasing Braking and Amortization Forces during the Countermovement Jump Does Not Necessarily Improve Jump Height

**DOI:** 10.5114/jhk/190451

**Published:** 2024-09-26

**Authors:** Daichi Nishiumi, Norikazu Hirose

**Affiliations:** 1 Graduate School of Sport Sciences, Waseda University, Saitama, Japan.; 2 Faculty of Sport Sciences, Waseda University, Saitama, Japan.

**Keywords:** ground reaction force-time curve, force-velocity relationship, rate of force development, countermovement strategy, kinetics

## Abstract

This study aimed to investigate the acute effects of altering the braking rate of force development (B-RFD) and amortization force (Amf) during countermovement jumps (CMJs) on CMJ height. Nineteen healthy men and women with training experience participated, performing six CMJ variations at different velocities (preferred and fast) and depths (knee angles: 60°, 90°, and 120°). The measured variables included CMJ height, B-RFD, Amf, and impulses during the early and latter halves of the concentric phase (EI and LI, respectively). A two-way analysis of variance was employed, along with a correlational analysis of the rates of change for each variable. Significant velocity and depth effects were observed for B-RFD and Amf (p < 0.05). However, there was no significant velocity effect on CMJ height. No significant correlations were observed between the rates of change in B-RFD and Amf with CMJ height. Additionally, a high or a very high correlation (r ≥ 0.67) was observed between the rate of change in B-RFD and Amf with the rate of change in EI, while a moderate negative correlation (r = −0.43 to −0.53) was found between the rate of change in EI and LI. These findings suggest that improvements in B-RFD and Amf were associated with improvements in EI, while improvements in EI led to a reduction in LI, and consequently, improvements in B-RFD and Amf were not associated with an increase in CMJ height. In other words, improvements in B-RFD and Amf did not necessarily contribute to improvements in CMJ height.

## Introduction

Improving jump performance has long been a key focus area in competitive sports. Studies have demonstrated that athletes competing at higher levels in volleyball, soccer, and basketball tend to achieve greater jump height (Haugen et al., 2012; Sattler et al., 2015; Spiteri et al., 2019 Spieszny et al., 2022). Moreover, jump height has been found to be related to both sprinting (Loturco et al., 2015; Maćkała et al., 2015) and the ability to change direction quickly (Hernández-Davó et al., 2021).

Newton’s second law states that jump height is determined by the net impulse generated during the concentric phase of the movement. An effective strategy to achieve this is to use countermovement. For instance, the countermovement jump (CMJ) involves countermovement immediately before the concentric phase, resulting in a higher jump than a squat jump performed without countermovement (Bobbert et al., 1996). The use of countermovement has been found to increase the force during the braking phase (the phase of deceleration in downward velocity during countermovement) just before the concentric phase of a jump. This results in a greater force being exerted from the amortization moment (transition moment from the braking to the concentric phase) to the early concentric phase, which, in turn, contributes to a higher total concentric net impulse and, thus, a greater jump height (Cormie et al., 2010). However, it remains debatable whether an increase in the forces during the braking phase and amortization moment due to countermovement necessarily leads to an improvement in jump height. Enhancing the force during the braking phase implies an increase in the rate of force development during the braking phase (B-RFD: the rate of change of the ground reaction force from the beginning to the end of the braking phase during the CMJ, Technical error of measurement [TEM]: 433 N/s), as the braking phase impulse is determined by the impulse during the unloading period. Additionally, improving the B-RFD is closely related to enhancing the amortization force (Amf, TEM: 51.9 N) (Nishiumi and Hirose, 2023). However, there is still a lack of consensus regarding the relationship between CMJ height (TEM: 0.012 m) and the B-RFD (Nishiumi et al., 2023). Some studies have reported an association between the B-RFD and CMJ height (Laffaye et al., 2014; McHugh et al., 2021), while others have not (Merino-Muñoz et al., 2020; Nishiumi and Hirose, 2023). Furthermore, a report indicated that Amf does not correlate with CMJ height (Nishiumi and Hirose, 2023). One possible explanation for this inconsistency is that the reduced force exertion in the latter half of the concentric phase may have offset the force increase in the early phase (Nishiumi and Hirose, 2023). This may be because an increase in force during the early phase leads to a faster body center-of-gravity velocity in the latter half of the concentric phase. As a result, faster muscle contractions may be required, which can have a negative impact on force exertion in the latter half of the phase due to the force-velocity relationship of the muscles (Nishiumi and Hirose, 2023). This suggests that simply increasing the B-RFD and Amf may not necessarily result in a greater jump height.

Another potential reason for the lack of scientific consensus is the absence of unified countermovement strategies. A recent systematic review investigating the variables during the CMJ downward phase and their relationship to CMJ height showed that most previous studies did not specify the countermovement strategies (Nishiumi et al., 2023). The velocity and depth of the countermovement have been found to influence variables during the downward phase (Pérez- Castilla et al., 2021), making it difficult to accurately investigate their associations without proper specifications. Pérez-Castilla et al. (2021) compared variables during the CMJ downward phase by altering the countermovement velocity and depth. However, they did not investigate the relationship between the B-RFD, Amf, and CMJ height. Furthermore, the associations of the B-RFD and Amf with CMJ height have only been examined through correlation studies (Nishiumi et al., 2023). To our knowledge, no studies have investigated this relationship through acute interventions.

Given the lack of clarity regarding the effects of the B-RFD and Amf due to intervention in countermovement strategy on CMJ height, this study aimed to investigate the effects of acute changes in the B-RFD and Amf on CMJ height. The B-RFD and Amf are believed to be influenced by both the velocity and depth of the squatting motion during countermovement (Pérez-Castilla et al., 2021). Therefore, in this study, we attempted to acutely manipulate the B-RFD and Amf by altering the velocity and depth of the countermovement. In addition, we compared the early and latter halves of the concentric impulse under different conditions and investigated the relationship between the increase in the impulse during the early half and the decrease in the impulse during the latter half. The hypothesis was that while changes in the B-RFD and Amf would not affect CMJ height, the increase in the impulse during the early half of the concentric phase would be offset by a decrease in the impulse during the latter half.

## Methods

### Participants

Nineteen healthy adult men and women with at least one year of training experience (2 to 3 weight training sessions per week on average) in exercise participated in this study (males: *n* = 11; age, 24 ± 2 years; body mass, 69.8 ± 6.0 kg; body height, 172.5 ± 4.2 cm; females: *n* = 8; age, 23 ± 3 years; body mass, 61.6 ± 8.6 kg; body height, 160.3 ± 5.1 cm; [means ± standard deviations]). None of the participants had musculoskeletal disorders. Before the commencement of the study, we used a priori power analysis for a two-way analysis of variance conducted using G*Power software version 3.1 (Heinrich-Heine-Universität Düsseldorf, North-Rhine Westphalia, Germany) to determine the number of participants that should be included in the experiment; this number was determined to be 18. The statistical significance level, statistical power, and effect size were set at 0.05, 0.8, and 0.25, respectively. The effect size was determined based on a previous study (Nishiumi and Hirose, 2023). This study was approved by the Waseda University Institutional Review Board (approval code: 2021-089; approval date: 31 July 2021) and was conducted in accordance with the principles of the Declaration of Helsinki. Prior to obtaining written informed consent from the participants using a university-approved document, comprehensive information regarding the potential benefits and risks of the investigation was provided to them through oral and written means. This ensured that the participants clearly understood the study before providing their consent.

### Procedures

The study was conducted over two days, with both days beginning with a combination of free stretching and prescribed dynamic stretching. On day 1, a familiarization session with the CMJ was conducted. Six different CMJs (60-preferred, 60-fast, 90-preferred, 90-fast, 120-preferred, and 120-fast) were performed by combining three different depths (knee joint angles of 60°, 90°, and 120°) and two different velocities (self-selected velocity and as fast as possible). On day 2, participants performed two attempts of each of the six types of CMJs on a force plate. The time interval between the first and second days was set to be at least 48 h, but no more than 1 week.

### Measurement of the Exercise

The CMJs were conducted using only the body weight, and the hands were placed on the hips during movement. To determine the depth of the squat, a timing gate was placed beneath the buttocks to emit a sound when the knee joint was flexed to 60°, 90°, or 120° (supplementary file 1). The knee joint angle was measured using a manual goniometer with a line connecting the greater trochanter, lateral epicondyle of the femur, and lateral malleolus defining the angle. The velocity conditions for squatting were “fast” (as fast as possible) and “preferred” (self-selected velocity). Due to the participants having some difficulty performing the countermovement at a specified velocity (Pérez-Castilla et al., 2021), the study considered both their maximum voluntary velocity and self-selected velocity. On day 1, height of the timing gate for each participant was recorded for the three different squat depths. Each of the six types of CMJs was practiced for a minimum of three repetitions to enable the participants to become accustomed to them. On day 2, the six types of jumps were measured in random order. Each jump was performed twice to assess the reliability of the measurements. Prior to each trial, under the fast condition, participants were instructed to “squat as fast as possible and jump as high as you can”, while under the preferred condition, they were instructed to “squat at your own pace and jump as high as you can”. In cases where the desired position of the recoil depth was not reached (i.e., when the timing gate did not emit a sound), it was considered a failed attempt, and the measurement was repeated. A rest period of at least 30 s was provided between each attempt.

### Measurement Equipment and Data Analysis

The CMJ measurements were performed using two force plates (Hawkin Dynamics, Westbrook, Maine, USA) at a sampling rate of 1000 Hz (the TEM for jump height, B-RFD, and Amf were 0.012 m, 439.4 N/s, and 51.5 N, respectively). The obtained waveform was processed with a 50-Hz low-pass filter. From the obtained force-time curves, the following variables were computed: jump height, B-RFD, Amf, downward velocity, countermovement depth, and the early and latter halves of the concentric net impulse during the CMJ. To obtain the body weight, each jump was preceded by 1 s of static standing on the force plate. The velocity of the body’s center of mass (COM) was determined using the trapezoidal integration rule (Linthorne, 2001). To calculate the net force, the body weight was subtracted from the vertical ground reaction force (GRF). The resulting value was divided by the body mass to obtain the acceleration. The COM velocity and displacement were calculated by integrating the obtained acceleration and COM velocity, respectively. The phases of the CMJ were determined based on previous research (Harry et al., 2020): the beginning of the unloading phase was defined as the point at which the vertical GRF decreased by 2.5% of the body weight; the end of the unloading and beginning of the yielding phases were marked by the point at which the GRF reached its minimum value; the end of the yielding and the beginning of the braking phases were marked by the point at which the COM velocity reached its minimum value; the end of the braking and the beginning of the concentric phases was marked by the point at which the COM velocity reached zero; and the end of the concentric phase was identified when the GRF dropped below 20 N. The B-RFD during the CMJ was determined by dividing the change in force at the beginning and the end of the braking phase by the duration of the braking phase. The Amf represented the force at the beginning of the concentric phase, and the downward velocity was defined as the maximum negative velocity during the countermovement. Jump height was calculated using the following formula:


$$h={\text{Vto}}^{2}/2g,$$

where h represents jump height, Vto is the vertical COM take-off velocity, and g is the acceleration due to gravity.

The early half of the concentric net impulse (EI) was defined as the impulse from 0% to 50% of the concentric phase, considering the entire duration of the concentric phase to be 100%. The latter half of the concentric net impulse (LI) was obtained by subtracting the EI from the total concentric net impulse.

### Statistical Analyses

The results are presented as means ± standard deviations. Statistical analyses were performed using the social sciences software package (IBM SPSS Statistics version 28). To assess reliability, the coefficient of variation (CV) was calculated for both trials. Acceptable reliability was defined as a CV < 10% (Cormack et al., 2008). The significance level was set at *p* < 0.05.

A two-way repeated-measures analysis of variance (ANOVA) and post-hoc Bonferroni tests were performed to compare the results among the six types of the CMJ. The effect size was calculated using partial eta-squared (*η*^2^) (small, > 0.01; moderate, > 0.06; and large, > 0.138).

We calculated the rate of changes in CMJ height, B-RFD, Amf, EI, and LI between the “preferred” and “fast” conditions. To analyze the correlation between the rates of change of variables, the Pearson’s correlation coefficient (*r*) or the Spearman’s correlation coefficient (*ρ*) was used. The interpretations were as follows: trivial (<0.1), small (> 0.1), moderate (> 0.3), high (> 0.5), very high (> 0.7), and practically perfect (> 0.9) (Hopkins et al., 2009).

## Results

**Figure 1:**
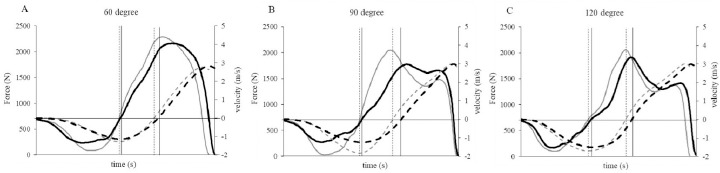
. Typical force-time curves for CMJs under each condition. The solid black and gray lines represent the ground reaction forces under the “preferred” and “fast” conditions, respectively. The dashed black and gray lines represent the center of mass velocity under the “preferred” and “fast” conditions, respectively. The intervals between the vertical solid and dashed lines represent the braking phases of the “preferred” and “fast” conditions, respectively. CMJ, countermovement jump

All variables showed a CV of < 10%, demonstrating their reliability (Table 1). Typical force-time curves for CMJs under each condition are shown in Figure 1. The results for CMJ height, B-RFD, Amf, downward velocity, countermovement depth, EI, and LI are presented in Table 2. In the downward velocity, a significant velocity effect was observed, and post-hoc tests revealed that under all angular conditions, the fast condition exhibited significantly higher negative velocities. In terms of the countermovement depth, a significant angular effect was observed, and post-hoc tests revealed significant differences between the 60° condition and both the 90° and 120° conditions, as well as between the 90° and 120° conditions. Additionally, no significant velocity effect was found, and there were no significant differences in countermovement depth between velocity conditions. In the B-RFD and Amf, both velocity and angle effects were observed, with the B-RFD also showing an interaction effect. CMJ height showed a significant angle effect, but no significant velocity effect. However, both the B-RFD and Amf exhibited significant angle and velocity effects, with the B-RFD exhibiting an interaction effect. Both the EI and LI showed significant velocity effects, and LI also demonstrated an angle effect. The correlations between the rates of change in the variables are presented in Tables 3–5. Under all angle conditions, no significant correlation was found between the rates of change in CMJ height and in the B-RFD and Amf.

**Table 1:** CV values for the variables in the results.

CV (%)	60-preferred	60-fast	90-preferred	90-fast	120-preferred	120-fast
CMJ height	2.0 ± 1.7	2.0 ± 2.7	2.3 ± 1.9	2.6 ± 2.1	2.1 ± 1.4	1.8 ± 1.8
B-RFD	3.8 ± 2.9	5.9 ± 4.7	6.9 ± 5.1	4.3± 4.3	7.1 ± 6.2	4.9 ± 5.0
Amf	2.0 ± 1.4	2.1 ± 1.5	2.8 ± 1.7	1.7 ± 1.3	2.0 ± 1.4	1.9 ± 1.6
Downward velocity	6.2 ± 4.7	2.6 ± 1.8	5.6 ± 4.7	3.4 ± 2.6	4.7 ± 2.6	3.4 ± 2.7
Countermovement depth	3.7 ± 3.4	4.6 ± 3.1	2.8 ± 2.5	3.8 ± 3.2	2.5 ± 1.8	2.6 ± 2.4
EI	1.4 ± 1.4	1.4 ± 1.2	1.8 ± 1.4	2.3 ± 1.5	1.8 ± 1.4	1.3 ± 1.0
LI	2.9 ± 3.1	3.7 ± 2.5	3.3 ± 2.9	4.1 ± 4.5	2.8 ± 2.4	2.6 ± 2.3

CV: coefficient of variation, CMJ: countermovement jump, B-RFD: braking rate of force development, Amf: amortization force, EI: early half of the concentric impulse, LI: late half of the concentric impulse

**Table 2:** Results of the two-way analysis of variance.

		Angle			Velocity effect			Angle effect		Interaction	
	Velocity	60°	90°	120°	*p* values	Partial *η*^2^	*p* values		Partial *η*^2^	*p* values	Partial *η*^2^
CMJ height (m)	Preferred	0.32 ± 0.05†‡	0.35 ± 0.06	0.36 ± 0.06	0.47	0.03	< 0.01		0.69	0.10	0.12
	Fast	0.31 ± 0.04†‡	0.35 ± 0.06	0.36 ± 0.06							
B-RFD (N/s)	Preferred	7534 ± 1957†‡	5645 ± 1516	5128 ± 1945	< 0.01	0.76	< 0.01		0.74	< 0.01	0.33
	Fast	16266 ± 2101*†‡	6868 ± 1373*	6614 ± 1847*							
Amf (N)	Preferred	1657 ± 252.5	1589 ± 245.4	1616 ± 280.8	< 0.01	0.76	< 0.01		0.30	0.19	0.02
	Fast	1827 ± 247.6*†	1680 ± 222.1*‡	1756 ± 286.5*							
Downward velocity (m/s)	Preferred	−1.06 ± 0.20†‡	−1.40 ± 0.16‡	−1.52 ± 0.25	< 0.01	0.69	< 0.01		0.88	0.40	0.05
	Fast	−1.22 ± 0.11*†‡	−1.59 ± 0.10*‡	−1.75 ± 0.12*							
Countermovement depth (m)	Preferred	−0.23 ± 0.04†‡	−0.35 ± 0.03‡	−0.44 ± 0.04	0.22	0.08	< 0.01		0.96	0.03	0.17
	Fast	−0.21 ± 0.03†‡	−0.35 ± 0.04‡	−0.44 ± 0.03							
EI (Ns)	Preferred	107.5 ± 17.7	106.6 ± 18.8	106.8 ± 19.4	< 0.01	0.64	0.33		0.06	0.09	0.14
	Fast	110.4 ± 17.4*	108.6 ± 19.0*	111.6 ± 19.7*							
LI (Ns)	Preferred	58.3 ± 10.7†‡	66.4 ± 10.9	67.1 ± 11.8	< 0.01	0.73	< 0.01		0.61	0.16	0.01
	Fast	52.8 ± 9.4*†‡	63.0 ± 11.9*	63.1 ± 11.4*							

CMJ: countermovement jump, B-RFD: braking rate of force development, Amf: amortization force, EI: early half of the concentric impulse, LI: late half of the concentric impulse. *: significant difference from the preferred condition (p < 0.05), †: significant difference from the 90° condition (p < 0.05), ‡: significant difference from the 120° condition (p < 0.05)

**Table 3:** Correlation coefficient between the rate of change under the “preferred” and “fast” conditions at 60°

%Δ	CMJ height	B-RFD	Amf	EI	LI
CMJ height	1	0.29	0.44	0.50*	0.44
B-RFD		1	0.90*	0.71*	−0.49*
Amf			1	0.87*	−0.54*
EI				1	−0.47*
LI					1

CMJ: countermovement jump, B-RFD: braking rate of force development, Amf: amortization force, EI: early half of the concentric impulse, LI: late half of the concentric impulse, *: p < 0.05, the gray area represents the Spearman’s rank correlation coefficient

**Table 4:** Correlation coefficient between the rate of change under the “preferred” and “fast” conditions at 90°.

%Δ	CMJ height	B-RFD	Amf	EI	LI
CMJ height	1	−0.02	−0.09	0.51*	0.33
B-RFD		1	0.97*	0.681*	−0.49*
Amf			1	0.67*	−0.51*
EI				1	−0.53*
LI					1

CMJ: countermovement jump, B-RFD: braking rate of force development, Amf: amortization force, EI: early half of the concentric impulse, LI: late half of the concentric impulse, *: p < 0.05, the gray area represents the Spearman’s rank correlation coefficient

**Table 5:** Correlation coefficient between the rate of change under the “preferred” and “fast” conditions at 120°.

%Δ	CMJ height	B-RFD	Amf	EI	LI
CMJ height	1	0.31	0.29	0.34	0.56*
B-RFD		1	0.93*	0.74*	−0.37
Amf			1	0.78*	−0.37
EI				1	−0.43
LI					1

CMJ: countermovement jump, B-RFD: braking rate of force development, Amf: amortization force, EI: early half of the concentric impulse, LI: late half of the concentric impulse, *: p < 0.05, the gray area represents the Spearman’s rank correlation coefficient

## Discussion

This study aimed to investigate the impact of the acute manipulation of the B-RFD and Amf on jump height. We also aimed to investigate the relationship between the increase in the early half of the concentric impulse and the changes in the latter half of the concentric impulse. The results of the two-way ANOVA revealed significant velocity and angle effects for the B-RFD and Amf; however, no velocity effect was observed for jump height. Additionally, in the correlation analysis of the rate of change, no significant correlation was observed between both the B-RFD and Amf and CMJ height. Furthermore, in the two-way ANOVA, the early and latter halves of the concentric impulse showed significant increases and decreases, respectively, under the fast condition. In the correlation analysis of the rate of change, a significant negative correlation was observed for the 60° and 90° conditions. These results suggest that improvements in the B-RFD and Amf did not contribute to an increased jump height, thus supporting our initial hypothesis.

In the present study, the B-RFD and Amf were varied by changing the velocity and depth of the countermovement in the CMJ. While previous research examined the CMJ with variations in squatting velocity and depth (Pérez-Castilla et al., 2021), to the best of our knowledge, the present study is the first to investigate the B-RFD and Amf and their impact on CMJ height in specifying the countermovement strategy. Furthermore, the results of the present study suggest that improvements in the B-RFD and Amf do not necessarily contribute to an increased CMJ height, which aligns with the findings of recent studies. Merino-Muñoz et al. (2020) reported no significant correlation between the B-RFD and CMJ height. In addition, in a cross-sectional study, Nishiumi and Hirose (2023) reported a very strong negative correlation between the B-RFD and Amf with the average force in the latter half of the concentric phase. Furthermore, Krzyszkowski et al. (2022) found that cueing improved the braking force, but there was no significant change in CMJ height.

These findings validate our results.

Based on the results of the two-way ANOVA, it was found that increasing the countermovement velocity significantly improved the B-RFD and Amf; however, there were no significant changes in jump height. Furthermore, there was an observed trend where the B-RFD decreased as the countermovement depth increased, while jump height showed an increasing trend. These findings indicate that improvements in the B-RFD and Amf do not contribute to jump height. These are likely influenced by the force-velocity relationship of the muscles. In the present study, the rates of change in the B-RFD and Amf showed a significantly high correlation with the rate of change in EI across all angle conditions. This suggests that an increase in the force during the concentric phase initiation of the CMJ can lead to an increase in the impulse during the early half of the concentric phase. The improvement in the impulse during the early half of the concentric phase can lead to an enhancement in the velocity of the COM ascent during the latter half of the concentric phase, necessitating further increases in muscle contraction velocity during the latter half. This potential effect can be attributed to the force-velocity relationship of the muscles, which may have a detrimental effect on muscle force generation. In fact, in the present study, a two-way ANOVA revealed that in terms of velocity conditions, EI and LI showed increases and decreases, respectively.

Furthermore, there were significant negative correlations between the rate of change in EI and LI for 60° and 90° conditions; however, under the 120° condition, there was a non-significant but moderate negative correlation. This indicates that increases in EI are associated with a decrease in LI. Nikolaidou et al. (2017) demonstrated, using ultrasound imaging, that the vastus lateralis muscle shortened during the concentric phase of the CMJ. Additionally, Nishiumi and Hirose (2023) reported a significant relationship between lower body muscle force characteristics in the force-velocity relationship and the GRF-time curve during jumping. These findings emphasize that the GRF-time curve regarding the CMJ is influenced by the force-velocity relationship of the muscles. In addition, Nikolaidou et al. (2017) reported that vastus lateralis force decreased with increasing contraction velocity due to the force-velocity relationship. Although the vastus lateralis is important for jump height because of the strong correlation that exists between the vastus lateralis thickness and jump height (Secomb et al., 2015), a decrease in the force of that muscle can have a significant negative effect. Furthermore, the gastrocnemius and hamstrings are also considered important for jump height (Wong et al., 2016). However, since the gastrocnemius and hamstrings, like the vastus lateralis, also have a force-velocity relationship (Baratta et al., 1995; Prietto and Caiozzo, 1989), a muscle force decrease occurred in the latter half of the concentric phase under the fast conditions. As a result, the decrease in lower extremity muscle force during the latter half of the concentric phase of the fast condition led to a 5–9% decrease in LI of the fast condition (compared to LI of the preferred condition). The improvement in the impulse during the early half of the concentric phase was almost offset by the decrease in the impulse during the latter half, resulting in no change in the total impulse and, consequently, no change in jump height.

Additionally, the present study examined the effects of varying countermovement depths. Changing the countermovement depth can influence variables during the descent phase, and by testing different depths, the present study aimed to verify whether similar results would be obtained across various depths. However, regardless of the depth of the condition, the relationships between the B-RFD, Amf, CMJ height, EI, and LI were consistent. Therefore, it can be inferred that factors contributing to the lack of improvement in CMJ height with the enhancement of the B-RFD and Amf remain consistent at any countermovement depth. On the other hand, a significant main effect of the countermovement depth was observed in jump height. As the countermovement depth increased, the push-off distance also increased, making it easier to generate a higher impulse, contributing to jump height (Sánchez-Sixto et al., 2018).

In practical applications, improving the B-RFD and Amf by quick squatting does not necessarily contribute to an increase in jump height. Therefore, allowing free countermovement is considered acceptable when aiming to improve jump height. However, it is generally known that, compared to squat jumps performed without countermovements, the CMJ typically yields higher jump heights (Bobbert et al., 1996). This is believed to be influenced by the muscle slack (Van Hooren and Zolotarjova, 2017). Therefore, it is necessary for the B-RFD to be sufficient in order to eliminate muscle slack.

Regarding the limitations of the present study, it should be noted that there were only three conditions for the knee angle and two conditions for the velocity during the countermovement. Therefore, it remains unclear whether similar results can be obtained at other depths or velocities. Further investigations through simulation studies or experiments under various conditions are required to validate and generalize our findings.

In conclusion, the present study demonstrates that neither the B-RFD nor the Amf necessarily contributes to vertical jump height. One possible reason for this is that improvement in the B-RFD and Amf leads to an increase in the impulse during the early half of the concentric phase; however, this is offset by a decrease in the impulse during the latter half of the concentric phase due to the muscle force-velocity relationship. As a result, there would be no change in the total impulse, leading to no significant contribution to vertical jump height. As a practical application, squatting quickly may not necessarily contribute to jump height, but squatting to a depth of 90 to 120 degrees of knee flexion may potentially increase jump height.

## Supplementary file 1

Examples of the depths under conditions of knee flexion angles of 60°, 90°, and 120°. A, B, and C represent the depths under the conditions of knee flexion angles of 60°, 90°, and 120°, respectively.
